# Drug discovery in leishmaniasis using protein lipidation as a target

**DOI:** 10.1007/s12551-021-00855-0

**Published:** 2021-11-04

**Authors:** James A. Brannigan, Anthony J. Wilkinson

**Affiliations:** Structural Biology Laboratory, Department of Chemistry, York Biomedical Research Institute, University of York, York, YO10 5DD UK

**Keywords:** *N*-myristoyltransferase, Protein structure, Neglected tropical disease, Leishmaniasis inhibitor discovery

## Abstract

The leishmaniases are infectious diseases caused by a number of species of obligate intracellular protozoa of the genus *Leishmania* with disease manifesting as cutaneous, mucocutaneous and visceral forms. Despite being endemic in more than 80 countries and its being the cause of high morbidity and mortality, leishmaniasis remains a neglected tropical disease. Chemotherapy is the frontline treatment, but drugs in current use suffer from toxic side effects, difficulties in administration and extended treatment times — moreover, resistance is emerging. New anti-leishmanial drugs are a recognised international priority. Here, we review investigations into *N*-myristoyltransferase (NMT) as a potential drug target. NMT catalyses the co-translational transfer of a C_14_ fatty acid from myristoyl-CoA onto the N-terminal glycine residue of a significant subset of proteins in eukaryotic cells. This covalent modification influences the stability and interactions of substrate proteins with lipids and partner proteins. Structure-guided development of new lead compounds emerging from high-throughput screening campaigns targeting *Leishmania donovani* NMT has led to the discovery of potent inhibitors which have been used to gain insights into the role of protein myristoylation in these parasites and to validate NMT as a drug target.

## Introduction

The leishmaniases are diseases caused by more than 20 different *Leishmania* parasite species (Burza et al. 2018; Sasidharan and Saudagar 2021). The three main forms of the disease are visceral leishmaniasis (VL) which is the most serious and often fatal if untreated, cutaneous leishmaniasis (CL) which is the most common and is associated with disfiguring skin lesions and mucocutaneous leishmaniasis in which the mucous membranes of the nose, mouth and throat are partially or totally destroyed. According to the World Health Organization (www.who.int/health-topics/leishmaniasis), more than 1 billion people live in areas where leishmaniasis is endemic, and it is estimated there are 30,000 and 1 million new cases, respectively, of VL and CL annually occurring mainly in South America, East Africa, the Middle East and the Indian subcontinent.

*Leishmania* parasites are transmitted to human and other mammalian hosts through the bite of a female phlebotomine sandfly when it takes a blood meal. The promastigote form of the parasite which is injected into the skin is taken up by macrophages, or other mononuclear phagocytes, where it becomes enclosed in a phagosome that fuses with lysosomes to form a parasitophorous vesicle (PV). In the PV, the parasite undergoes differentiation to the amastigote form. Amastigotes multiply in the PV before bursting from the cell to initiate new cycles of infection of cells located either in the immediate neighbourhood in the case of CL or after dissemination to distant tissues such as the liver, spleen and bone marrow in the case of VL.

**Fig. 1:**
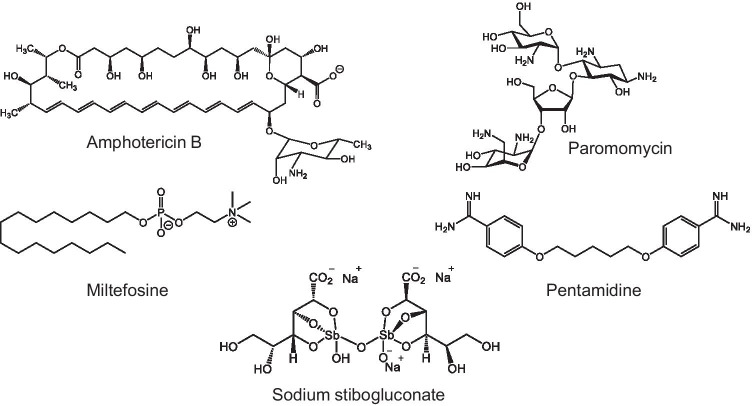
Chemical structures of a selection of clinically important anti-leishmanial drugs in current use

In the absence of a licenced vaccine, measures to counter leishmaniasis rely on chemotherapy (Burza et al. 2018; Sasidharan and Saudagar 2021). Current treatments include pentavalent antimonials such as sodium stibogluconate, amphotericin B, miltefosine, paromomycin and pentamidine (Fig. 1). Treatments suffer variously from the need for hospitalisation and monitoring, toxic side effects, prolonged treatment times and high costs. Moreover, poor diagnosis and complex co-infections diminish the effectiveness of therapy and the lack of oral formulations complicates delivery and reduces compliance. The effectiveness of therapy is further compromised by the frequency of relapse and the emergence of drug resistance.

The use of antimony (Sb)-containing compounds has dominated the treatment of leishmaniasis for a century or so despite the need for parenteral administration, toxic side effects and high costs. More recently, the natural product amphotericin B, an anti-fungal agent synthesised by certain *Streptomyces* species, has supplanted sodium stibogluconate as the first-line treatment of VL in many parts of the world. Its toxic side effects are considerably reduced when it is delivered as a liposomal formulation, AmBisome. However, it too has to be injected, and again, it is expensive. Miltefosine is a repurposed anti-cancer drug and an alkyl phosphocholine which can be orally administered for treatment of VL, though it is expensive. It too suffers from side effects, and significantly, it has been identified as a teratogen which limits its suitability. It has a long half-life in the body giving rise to concerns that resistance will emerge as the parasites are exposed to sub-lethal concentrations of the drug. Meanwhile, paromomycin, an aminoglycoside antibiotic which has been repurposed for the treatment of cutaneous leishmaniasis, is applied topically as an ointment. Pentamidine is an aromatic diamidine often used to treat CL which is thought to have a mitochondrial site of action. For all of the drugs, little is known of the target(s), the mechanism of action or the mechanism of resistance.

## N-myristoyltransferase and protein myristoylation in *Leishmania*

The enzyme N-myristoyltransferase (NMT) catalyses the transfer of the C_14_ fatty acid, myristate, from myristoyl-CoA onto the N-terminal glycine residue of a subset of cellular proteins (Meinnel et al. 2020). N-myristoylation of proteins typically takes place co-translationally following the action of methionine aminopeptidase on nascent peptides emerging from the exit tunnel of the ribosome. Lipidation has been shown to influence the stability of proteins and to determine their partitioning into membranes as part of protein trafficking and localisation. It can also shape their interactions with protein partners. NMT is a 50-kDa monomeric protein. There is a single NMT in protozoan parasites in contrast to the two, NMT1 and NMT2, present in the human host.

**Fig. 2:**
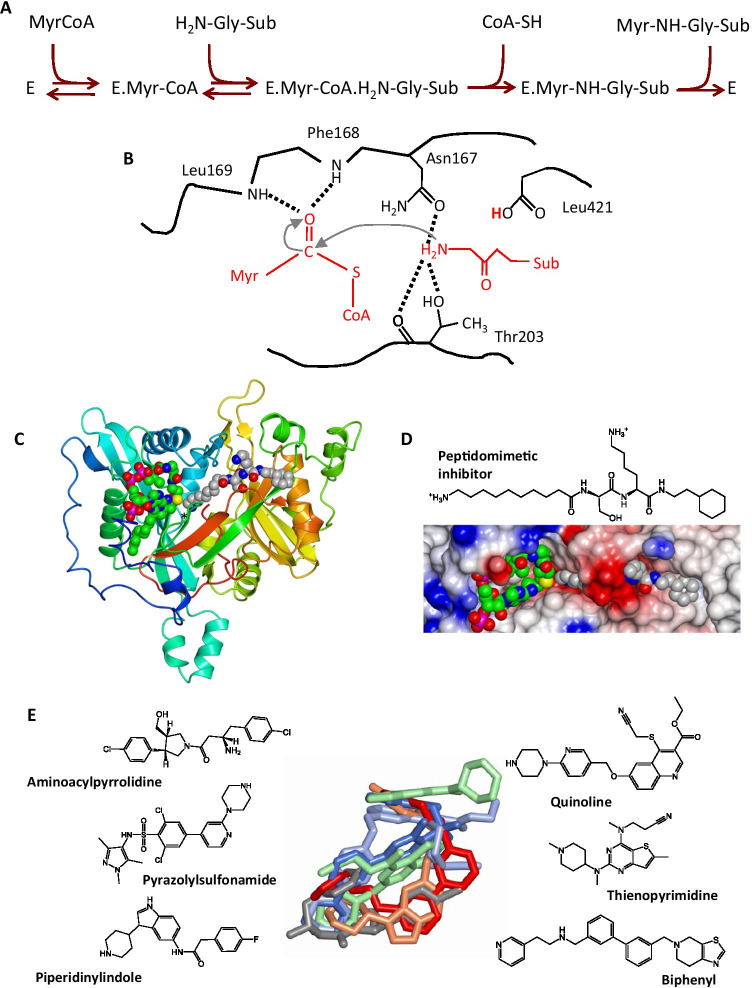
Structure and ligand binding to *Leishmania* NMT. **A** Scheme showing the ordered binding of substrates and the ordered release of products in the NMT-catalysed reaction. **B** Key step in the catalytic mechanism. Following binding of the substrates and deprotonation of the substrate protein’s α-amino group by the carboxylate of Leu421, there is nucleophilic attack of the amino group of the glycine on the carbonyl carbon of the thioester of myristoyl-CoA. **C** The crystal structure of the ternary complex of NMT from *L. major* (PDB code 4c7h) with myristoyl-CoA and a peptidomimetic inhibitor bound in the active site. The protein is represented as a ribbon colour-ramped from the N-terminus (blue) to the C-terminus (red). The C-terminus is additionally labelled with an asterisk. The ligands are shown in sphere representation with atoms coloured by type: carbon, grey for peptidomimetic and green for myristoyl-CoA; oxygen, red; nitrogen, blue; sulphur, yellow; phosphorus, magenta. **D** Lower, zoom view of **C** with electrostatic surface rendering of the protein molecule emphasising the substrate binding groove. Upper, chemical structure of the peptidomimetic inhibitor (N-(10-aminodecyl)-L-seryl-N-(2-cyclohexyl)-L-lysinamide). **E** Structures of inhibitor molecules described in the text. In the centre, these molecules are displayed following least squares superposition of the protein Cα atoms in the respective structures of the LmNMT-myristoyl-CoA inhibitor ternary complexes. The inhibitors occupy overlapping volumes in the peptide-binding cavity. The inhibitors are coloured: aminoacylpyrrolidine, grey; pyrazolyl sulfonamide, blue; piperidinylindole, coral; quinoline, ice blue; thienopyrimidine, green; biphenyl, red

The specificity and mechanism of the NMTs from *Candida albicans*, *Saccharomyces cerevisiae* and most recently human NMT1 have been studied in great detail (Bhatnagar et al. 1998, 1994; Dian et al. 2020; Lodge et al. 1994; Rudnick et al. 1991), and it is assumed that knowledge of these enzymes carries over to the enzymes from parasites. Although the sequences of the parasite NMTs share only 40–45% sequence identity with their fungal and human orthologues, the sequence conservation is much higher in the active site. Reaction proceeds via a compulsory order mechanism (Fig. 2A) with binding of myristoyl-CoA preceding that of the substrate protein, and following acyl transfer via a nucleophilic addition–elimination reaction (Fig. 2B), CoA release precedes dissociation of the acylated protein (Rudnick et al. 1991). The structures of NMT from *L. donovani* and *L. major* (Brannigan et al. 2010; Frearson et al. 2010) feature a characteristic 12-stranded twisted β-sheet which provides the base of an extended groove. The left-hand half of the groove as shown in Fig. 2C forms the binding site for myristoyl-CoA with the right-hand half forming a binding site for the amino terminal peptide segment of the substrate protein or, as in Figs. 2C and D, a peptidomimetic inhibitor (Olaleye et al. 2014). The α-carboxylate at the C-terminus of the protein resides at the heart of the structure. It plays a key role in catalysis, acting as a base to deprotonate the α-amino group on the protein substrate for nucleophilic attack on the carbonyl carbon of the thioester of myristoyl-CoA (Fig. 2B).

ADP-ribosylation factor-like proteins and the hydrophilic acylated surface proteins were among the first myristoylated proteins to be identified in *Leishmania* though it was anticipated that the repertoire of myristoylated proteins would be much broader (Denny et al. 2000; Sahin et al. 2008). The absence of a clear myristoylation consensus sequence hampered the identification of NMT substrates. A fuller repertoire has been defined by metabolic labelling in vivo with an alkyne-functionalised myristic acid. Downstream click chemistry was then used to couple the fatty acid–acylated proteins to an azide-capture reagent containing biotin and a fluorophore (Wright et al. 2014). Mass spectrometry of the enriched and gel-electrophoretically resolved protein set led to the identification of 30 high-confidence NMT substrates in *L. donovani* with roles in protein phosphorylation, protein transport and degradation. The role of myristoylation in diverse regulatory functions suggests that NMT is an essential enzyme in *Leishmania* and therefore a rational target for the development of new therapeutics targeting leishmaniasis. Supporting this notion, NMT has been shown to be essential in the protozoan parasites, *T. brucei*, *T. cruzi* and *P. falciparum*, using both genetic and chemical methods (Brannigan and Wilkinson 2016; Frearson et al. 2010; Herrera et al. 2016; Schlott et al. 2018; Wright et al. 2014).

## Discovery of *L. donovani* NMT inhibitors

The springboard to the discovery of inhibitors of NMT from *L. donovani* (LdNMT) was a high-throughput screen carried out with 150,000 selected compounds from the Pfizer Global Diversity Representative Set which included legacy compounds from anti-fungal NMT programmes (Bell et al. 2012). A scintillation proximity assay was carried out for LdNMT in parallel with a screen against NMT from *P. falciparum* with promising compounds subsequently assayed for activity against NMT1 and NMT2 from the human host. This led to the identification of four novel series of compounds with submicromolar IC_50_ values (0.1–1μM) against LdNMT and 10–700-fold selectivity over the human orthologues (Bell et al. 2012).

To guide the downstream medicinal chemistry campaign, crystal structures were determined of complexes of *L. major* NMT, which has 97% sequence identity to *L. donovani* NMT, with representative compounds from the four series (Fig. 2E) (Brannigan et al. 2014). These structures were compared with those of LmNMT complexes of a potent pyrazolyl sulphonamide inhibitor of *T. brucei* NMT (Frearson et al. 2010) and a quinoline inhibitor of *P. vivax* NMT (Goncalves et al. 2017) (Fig. 2E). In ternary complexes with myristoyl-CoA, these inhibitors occupy overlapping volumes in the peptide-binding groove.

**Fig. 3:**
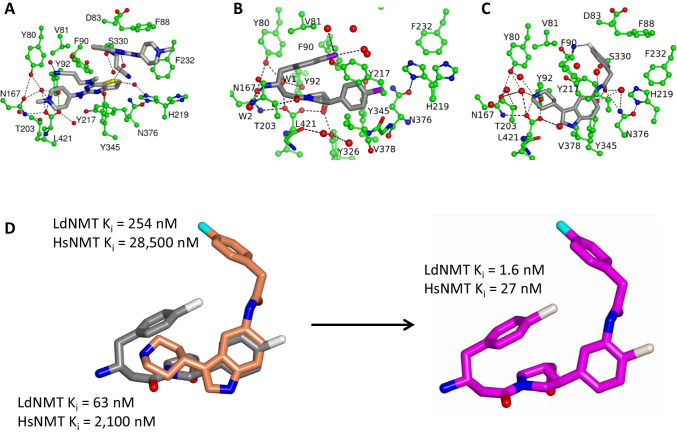
The structure of the inhibitor binding pocket and protein–inhibitor interactions. **A** The thienopyrimidine (PDB Entry, 4cgo), **B** the aminoacylpyrrolidine (4cgn) and **C** the piperidinylindole (4cgm) ligands are shown in cylinder representation with grey carbon atoms with the surrounding protein in ball-and-stick and green carbon atoms. The myristoyl-CoA cofactor, which is situated to the left of the ligand in each case, has been omitted for clarity. **D** Hybridisation of the binding modes of the aminoacylpyrrolidine (grey carbons) and piperidinylindole inhibitors (coral carbons) to generate a more potent hybrid NMT inhibitor (magenta carbons). Other atoms are coloured by type: oxygen, red; nitrogen, blue; chlorine, white; fluorine, cyan

The thienopyrimidine (Fig. 2E and 3A) inhibitor has an IC_50_ value of 0.25μM and tenfold selectivity over the human NMT isoform 1 (HsNMT1). Unexpectedly, two molecules occupy the binding site with their bicyclic aromatic rings stacking against each other (Bell et al. 2020; Brannigan et al. 2014). The higher affinity ligand (lower left in Fig. 3A) binds in a proximal site with its tertiary amine forming a salt-bridge to the main chain carboxylate of the enzyme’s C-terminal residue Leucine 421. The weaker binding ligand (upper right in Fig. 3A), as evidenced by its poorer associated electron density and its higher atomic temperature factors, is distally located (Brannigan et al. 2014). The piperidinylindole (Figs. 2E and 3C) has a *K*_i_ of 250 nM against LdNMT and > 100-fold selectivity against HsNMT1. In the binding site, this inhibitor wraps around the side chain of Phe90 with its two six-membered rings packing onto opposite faces of the aromatic ring of the side chain (Fig. 3C). The piperidine ring nitrogen forms an ion-pair with the α-carboxylate of the C-terminal residue Leu421.

The aminoacylpyrrolidine hit (Fig. 2E) was identified through analogue screening of the primary high-throughput screening hit explaining its low IC_50_ value of 0.08μM against LdNMT. It has a selectivity factor of ~ 80. The crystal structure of the aminoacylpyrrolidine complex resolved a stereochemical ambiguity (the two enantiomers exhibiting IC_50_ values of 0.025μM and 1.7μM) and revealed the inhibitor to be bound in a conformation that can be described as hydrophobically collapsed (Brannigan et al. 2014). The folding of the inhibitor directs its centrally located primary amine towards the myristoyl-CoA where it makes contacts with catalytically important residues and a conserved water molecule (Fig. 3B).

Comparison of the structures showed significant overlap in the volumes occupied by the aminoacylpyrrolidine and piperidinylindole inhibitors with overlap of one of the chlorophenyl moieties of the former with the indole ring of the latter (Brannigan et al. 2014; Hutton et al. 2014) (Fig. 3D). This suggested the possibility of synthesising a hybrid molecule. As shown in Fig. 3D, the resulting hybrid inhibitor exhibited a *K*_i_ of 1.6 nM for LdNMT which is 40-fold and 150-fold higher than the *K*_i_ values of the parent molecules. It can be seen in the crystal structure that the hybrid, or merged, inhibitor picks up on the binding mode of both parent molecules (Fig. 3D) such that all of the key interactions with the enzyme are conserved. This presents a nice example of this approach (Zhang et al. 2019). In this case, however, there is a trade-off as the gain in potency is offset by a significant loss of selectivity (selectivity factor reduced to 17) with respect to HsNMT (Hutton et al. 2014). The knowledge gained in these studies was later used in the development of potent inhibitors of human N-myristoyltransferase that block capsid assembly and replication of the common cold virus. In this study, rather than merging overlapping binding modes, fragments discovered to bind in neighbouring volumes were merged leading to spectacular gains in potency (Mousnier et al. 2018).

## Validation of NMT as a drug target in *Leishmania*

Gene deletion studies showed that it was possible to obtain parasites with single but not double knock-outs of the NMT gene in *L. donovani* promastigotes, effectively demonstrating that NMT is essential to parasites in this life cycle stage (Brannigan et al. 2010). For therapeutic purposes, it is necessary to kill the intracellular amastigotes, and therefore, it is important to establish that the target protein is essential to this life cycle stage. For NMT, this was established using a more elaborate plasmid shuffle approach (Paape et al. 2020). Here, parasites with both copies of the NMT gene deleted were generated following transfection with a plasmid harbouring a functioning copy of the NMT gene and a gene encoding thymidine kinase (TK). Upon treatment with the nucleoside analogue, ganciclovir, the presence of the TK gene provides a means of negative selection for the plasmid. This is because following TK-mediated phosphorylation of ganciclovir, the resulting nucleotide interferes with DNA strand elongation during replication, leading either to cell death or forcing expulsion of the plasmid. Ganciclovir treatment of mice that had established infections with amastigotes and later analysis of the spleen parasite burden and composition showed that NMT is indeed essential for the viability of intracellular amastigotes (Paape et al. 2020).

In a chemical validation approach, metabolic tagging of NMT substrates in both promastigotes and amastigotes was achieved using an alkyne mimetic of myristic acid as described above (Wright et al. 2015). As well as allowing definition of the complement of NMT substrates in the *L. donovani* life cycle stages, this methodology provided an assay of in vivo target engagement by NMT inhibitors. The study explained a disparity in the properties of a pair of pyrazole sulphonamide inhibitors related to the molecule shown in Fig. 2. Both exhibit similar (2.5-fold difference) nanomolar potencies in enzyme inhibition, but there is a 50-fold difference in their *EC*_50_ against ex vivo amastigotes. Using in vivo alkyne tagging, the study showed that parasite cell killing (*EC*_50_) correlated with inhibition of in vivo tagging, validating NMT as a drug target. However, even for the better acting inhibitor, the translation from in vitro enzyme inhibition (*K*_i_) to in vivo enzyme inhibition and cell killing (*EC*_50_) was poor (*EC*_50_/*K*_i_ = 25). The authors concluded that the inhibitor was not efficiently accessing its target.

In other work, a cosmid-based overexpression library screening approach was taken towards the pharmacological validation of a lead compound as a drug target in *L. donovani* (Corpas-Lopez et al. 2019). The study concerned a potent pyrazole sulphonamide inhibitor of LdNMT (*K*_i_ = 0.34 nM) that had only modest activity against *L. donovani* intracellular amastigotes (*EC*_50_ = 2.4μM). Parasites transfected with a high-coverage representative bar coded cosmid library were grown for 12 days in the presence of the inhibitor at a concentration twofold above the *EC*_50_. Isolation and sequencing of the cosmids maintained in the resistant population showed enrichment of fragments containing the gene encoding LdNMT consistent with specific targeting of the enzyme by the pyrazole sulphonamide. Confirming this interpretation, the overexpression of LdNMT in promastigotes led to a tenfold reduction in sensitivity to this inhibitor (Corpas-Lopez et al. 2019). In a complementary approach, thermal proteome profiling showed that the same inhibitor stabilises LdNMT against heat denaturation in parasite lysates, again suggesting on-target engagement (Corpas-Lopez et al. 2019). Consistent with these encouraging indicators, oral dosing of this compound in a mouse model of VL led to a 50% reduction in parasite burden.

## Conclusions and future prospects

The NMT inhibitors discovered by high-throughput screening approaches to date have been competitive ones which exclusively occupy the peptide-binding site rather than the fatty acyl-CoA binding site. Myristoyl-CoA binds to NMT with ~ 100-fold higher affinity (Johnson et al. 1994) than peptide substrates; moreover, its binding site is more highly conserved between the parasite and host NMTs. Fatty acyl-CoAs are shared substrates with other cellular enzymes which would present a specificity challenge. The latter concern could be overcome using a covalent inhibitor strategy as has been applied successfully in targeting the ATP binding sites of protein kinases (Zhao and Bourne 2018).

Despite the achievement of high potency inhibitors of NMT from *Leishmania* species by structure-guided medicinal chemistry and the development of sophisticated assays of NMT function and inhibition in vivo, we and others have been unable to design inhibitors that kill parasites inside macrophages at reasonable therapeutic doses. This is striking as many of the compounds generated in these studies exhibit on-target activity in *P. falciparum*-infected red blood cells and *P. berghei* infected mice and even in the targeting of human NMT1 as a strategy to counter human rhinovirus infection (Mousnier et al. 2018; Wright et al. 2014). The failure of our *Leishmania* NMT inhibitors may be due to their limited uptake by the amastigote stage parasites which bear glycoinositolphospholipid coats and reside within a parasitophorous vacuole in the host cell (Novozhilova and Bovin 2010). It may yet prove possible to introduce the right chemistry for parasite cell access into leads emerging from alternative screens.

To identify components of other post-translational modification systems with potential as drug targets, Mottram and co-workers have been systematically knocking out genes encoding protein kinases and protein ubiquitination/deubiquitination system factors in *L. mexicana*. They found scores of genes for which null mutants could not be generated and which are likely to be essential in the promastigote stage (Baker et al. 2021; Burge et al. 2020; Damianou et al. 2020). To allow identification of genes needed for differentiation to the human infectious parasite forms, their CRISPR-Cas9 repair cassettes included a unique 12-nucleotide barcode. Following pooling of sets of null mutants and growth and differentiation under different regimes, the surviving parasite population was characterised using next-generation sequencing. This parallel phenotyping assay revealed a number of genes whose deletion leads to strong defects in amastigote differentiation, macrophage infectivity and capacity to survive in mice following footpad injection (Baker et al. 2021; Burge et al. 2020; Damianou et al. 2020). These genes warrant further investigation as promising future protein targets for anti-leishmanial drug discovery.
